# Draft Genome Sequence of *Klebsiella pneumoniae* Isolate PR04

**DOI:** 10.1128/genomeA.00418-13

**Published:** 2013-08-15

**Authors:** M. H. Zulkifli, L. K. Teh, L. S. Lee, Z. A. Zakaria, M. Z. Salleh

**Affiliations:** Integrative Pharmacogenomics Institute (iPROMISE), Faculty of Pharmacy, Universiti Teknologi MARA (UiTM) Malaysia, Bandar PuncakAlam, Selangor DarulEhsan, Malaysiaa; Department of Biomedical Science, Faculty of Medicine and Health Sciences, University Putra Malaysia (UPM), Serdang, Selangor, Malaysiab

## Abstract

*Klebsiella pneumoniae* PR04 was isolated from a patient hospitalized in Malaysia. The draft genome sequence of *K. pneumoniae* PR04 shows differences compared to the reference sequences of *K. pneumoniae* strains MGH 78578 and NTUH-K2044 in terms of their genomic structures.

## GENOME ANNOUNCEMENT

*Klebsiella pneumoniae* is a Gram-negative bacterium that causes various hospital- and community-acquired infections, such as bacteremia (1, 2), pneumonia (3), urinary tract infections (UTIs) (4), liver abscess, endophthalmitis, and meningitis (5, 6). The strains of *K. pneumoniae* in different geographical areas may vary in terms of pathogenicity and resistance towards antibiotics (1), and therefore it is of great interest to uncover the genome sequences of the isolate we obtained from the patient in our study. The genetic determinants underlying the virulence and pathogenicity of the local strain might be different from those of other isolated strains.

Here, we report the draft genome sequence of *K. pneumoniae* PR04, isolated from a patient in a local hospital in Malaysia.

*K. pneumoniae* PR04 was identified using Gram stain and biochemical tests. For biochemical tests, the API 20E kit (bioMérieux SA Co., France) was used to confirm the identity of the bacterium. The genome was sequenced using GAIIx (Illumina, San Diego, CA) at the Pharmacogenomics Centre (PROMISE), Faculty of Pharmacy, Universiti Teknologi MARA (UiTM), Malaysia. The quality of the sequencing output was determined using FastQC. Data were generated from a paired-end 101-bp run. The sequencing produced an output of 6,271,798 reads with 78× coverage. FastQC revealed the average G+C content of this sample to be about 57.4%, while both reference genomes, *K. pneumoniae* MGH 78578 and NTUH-K2044, have 57.2% and 57.4% G+C content, respectively. After trimming, 6,228,844 reads were produced with 99.32% of trimming efficiency.

The reads were assembled (*de novo* and mapped to references) using the CLC bio Genomic Workbench (CLC bio, Cambridge, MA). A total of 300 contigs were produced that covered a total genome size of 5,290,141 bp. The contigs were mapped against two reference sequences, *K. pneumoniae* MGH 78578 and NTUH-K2044, using CLC bio. Of the reads, 82% matched the sequences of *K. pneumoniae* NTUH-K2044, and 84.7% of reads matched the sequences of *K. pneumoniae* MGH 78578.

Scaffolds were generated using Optimal Synteny Layout of unfinished Assemblies (OSLay) (7) and were remapped against the raw sequence (101 bp) to validate the assembly result. OSLay showed that 41 out of 300 contigs were not part of the scaffolds. The total number of gaps was 85. The annotation and comparative analysis were performed using Rapid Annotations using Subsystems Technology (RAST) (8). The scaffolds of *K. pneumoniae* PR04 were annotated to have 564 subsystems, 4,738 coding sequences, 4,794 annotated genes, and 55 RNAs; 587 subsystems, 4,953 coding sequences, 5,065 protein-coding genes, and 111 RNAs were annotated in *K. pneumoniae* MGH 78578. As for *K. pneumoniae* NTUH-K2044, 583 subsystems, 4,873 coding sequences, 4,985 genes, and 111 RNAs were annotated.

### Nucleotide sequence accession numbers.

The genome sequence of *K. pneumoniae* PR04 has been deposited in DDBJ/EMBL/GenBank under the accession no. AOPN00000000. The version described in this paper is version AOPN02000000.
